# Allelic imbalances of chromosomes 8p and 18q and their roles in distant relapse of early stage, node-negative breast cancer

**DOI:** 10.1186/bcr1349

**Published:** 2005-11-02

**Authors:** Aki Morikawa, Tanisha Y Williams, Luc Dirix, Cecile Colpaert, Michael Goodman, Robert H Lyles, Diansheng Zhong, Wei Zhou

**Affiliations:** 1 Department of Hematology and Oncology, Winship Cancer Institute, Emory University School of Medicine, Atlanta, GA, USA; 2 Department of Epidemiology, School of Public Health, Emory University, Atlanta, GA, USA; 3 Department of Oncology, General Hospital Saint-Augustinus, Antwerp, Belgium; 4 Department of Pathology, University Hospital Antwerp, Edegem, Belgium; 5 Department of Biostatistics, Rollins School of Public Health of Emory University, Atlanta, GA, USA

## Abstract

**Introduction:**

Identification of breast cancer patients at risk for postoperative distant relapse is an important clinical issue. Existing pathological markers can predict disease recurrence only to a certain extent, and there is a need for more accurate predictors.

**Methods:**

Using 'counting alleles', a novel experimental method, we determined allelic status of chromosomes 8p and 18q in a case-control study with 65 early stage, node negative, invasive ductal carcinomas (IDCs). The association between allelic imbalance (AI) of both chromosomal markers and distant relapses was examined.

**Results:**

Eighty percent of tumors contained 8pAI and sixty-eight percent of tumors contained 18qAI. However, none of the tumor samples retained both chromosome 8p and 18q alleles. More importantly, tumors with 8pAI but not 18qAI were more likely to have distant relapse compared to tumors with 18qAI but not 8pAI.

**Conclusion:**

Our finding suggests that differential allelic loss of chromosomes 8p and 18q may represent subtypes of early stage IDC with different tumor progression behaviors.

## Introduction

Breast cancer is the most frequently diagnosed neoplasia and the second leading cause of cancer deaths in women world-wide [1]. The majority of all new breast cancer cases have early stage tumors without lymph node involvement [2]. Based on the current clinical and pathological prognostic indicators such as Tumor-Nodes-Metastases (TNM) staging, HER2/neu and hormonal receptor status, many early stage breast cancer patients receive unnecessary adjuvant treatment after the removal of a primary tumor [3]. As these therapies are associated with various side effects, identifying those who will be at a higher risk of distant relapse, and would thus most benefit from these therapies, is an important issue.

Much of the recent effort in the search for new predictors to improve prognostic ability has been directed towards the identification of genetic biomarkers. For example, cytogenetic studies have shown that the presence of an abnormal chromosome complement (aneuploidy) is associated with high tumor grade and invasive behavior, as well as with eventual disease progression. As the molecular basis of aneuploidy is allelic imbalance (AI), many studies have attempted to examine the association between the AI of individual chromosomes and disease progression [4]. The results of studies have been inconsistent, however, partly due to technical problems associated with existing methods of AI analysis of clinical specimens [5].

To address common difficulties associated with the AI analysis of primary tumors, we have recently developed a technique called 'counting alleles' that is specifically designed for the analysis of archived clinical specimens [6,7]. This method is quantitative and is not susceptible to PCR amplification bias or DNA degradation. Specifically, allelic status was determined by evaluating single nucleotide polymorphic (SNP) markers. Genomic DNA is isolated from archived paraffin-embedded specimens and diluted to such an extent that single molecules can be individually amplified in separate PCR reactions; hundreds of PCR reactions were carried out for each specimen [8]. The detection of different alleles of each SNP locus is carried out with SNP-specific molecular beacons, which were included in the PCR mixture to allow a single-step reaction setup [9-11]. Allelic status of a sample is determined quantitatively by directly counting the number of each allele in a sample. Normal tissues and tumors without allelic loss will have approximately equal counts for both alleles. Tumor samples with allelic loss but containing normal tissue contamination will have different counts for each allele. The presence or absence of AI is determined by a statistical approach called the sequential probability ratio test, which can determine whether a specific primary tumor specimen contains AI with up to 50% normal tissue contamination. The successful application of the 'counting alleles' approach has been demonstrated in studies of colorectal cancer and prostate cancer [7,12-14]. Here, the 'counting alleles' approach is used to evaluate the risk factors for disease relapse in early-stage, node-negative breast cancer.

## Materials and methods

### Study population

The study population consisted of lymph-node negative breast cancer patients who had received loco-regional treatment, consisting of mastectomy or tumorectomy followed by radiation therapy for T1-2N0M0 breast cancer at the University Hospital Antwerp and the general hospital Saint-Augustinus, Antwerp, Belgium between 1986 and 1992. These patients had not received any type of systemic adjuvant therapy (hormonal or cytotoxic) and had undergone complete axillary lymph node dissection for nodal status assessment.

In this case-control study of invasive ductal carcinoma (IDC) patients, cases were defined as those presenting with distant radiologically confirmed relapse within five years of initial loco-regional treatment. Controls were randomly selected from patients who had no evidence of recurrence after more than five years based on routine physical examination, yearly mammography, routine biochemical analysis, chest X-ray, annual liver ultrasound and bone scan. The description of the original patient selection has been previously published by Colpaert *et al*. [15].

Twenty-four cases and forty-one controls who had enough tissue samples for genetic analysis and had IDC were analyzed in this study. Each patient was assigned a unique identifier, although no personal identifiers were used in the analysis for this study. Of the 65 patients, 59 were informative for at least one polymorphic marker on chromosome 8p, and 59 were informative for at least one polymorphic marker on chromosome 18q.

Data on clinical information and pathologic information were collected by an oncologist (LD) and a pathologist (CC) at the hospitals mentioned above. The details of the tumor characteristics and pathological assessments have been previously published by Colpaert *et al*. [15].

### DNA purification and 'counting alleles' analysis

A total of 20 sections were cut from each paraffin block for paired tumor and normal specimens and mounted on non-coated slides. The first one was cut at 5 microns, stained with hematoxylin and eosin, and routinely mounted. This section was used for the identification of tumor and normal cells from each sample. Sections 2 through 20 were cut at 12 microns and stained with hematoxylin and eosin; no coverslip was applied. Regions enriched in tumor cells were microdissected from these sections, and the normal tissues were also isolated from the paired normal slides as controls. Microdissected tissue samples were heated at 85°C for 20 minutes in 20 ml of 0.2 M NaOH/1 mM EDTA, and then neutralized with 40 mM Tris, pH 8.0, 1 mM EDTA (80 ml). The resultant tissue mixtures were separated in a microcentrifuge for 1 minute at about 12,000 × g and DNA was purified from the supernatants with a QIAquick Kit (Qiagen, Cat No.28104, Valencia, CA, USA)

The status of chromosome 8p and 18q alleles was determined using the counting alleles technique [7]. SNP markers on chromosomes 8p and 18q were retrieved from the National Center for Biotechnology Information SNP database [16], and modified from previous studies [13]. Forward and reverse PCR primers were designed for seven SNPs on chromosome 8p and five SNPs on chromosome 18q. The DNA sequences of the PCR primers and the molecular beacons have been reported previously [12]. DNA from the non-neoplastic breast tissues was used to identify an informative SNP for chromosomes 8p and 18q in each patient using digital PCR. Allelic status analysis was then carried out on both the normal and tumor DNA. A sequential probability ratio test was used to determine whether AI was present in the tumor tissue [7]. All normal DNA tested in this study contained balanced alleles, and all analyses of AI were performed in a blinded fashion using coded samples.

### Statistical analysis

Χ^2 ^tests and Fisher's exact tests were used to compare clinical and tumor characteristics and allelic status with early distant relapse. Association between allelic status and early relapse was evaluated using unconditional logistic regression with exact option. All the statistical analysis was carried out using the Statistical Analysis System version 8.2 (SAS Institute, Cary, NC, USA).

## Results

This study focuses on patients with IDC. The mean ages at diagnosis were 53 years (range 34 to 71 years) for cases and 56 years (range 27 to 78 years) for controls. Forty-six percent of the cases were pre-menopausal compared to thirty-six percent of the control group (p > 0.05). With regards to tumor characteristic, the cases and the controls differed significantly with regards to tumor grade, size, presence of necrosis and fibrotic foci, and angiogenesis score (Table 1).

AI of chromosomes 8p and 18q was detected in 80% (47/59) and 68% (40/59), respectively, of the IDCs studied. Individually, the allelic status of 8p and 18q was not significantly associated with distant relapse (Table 2). Combination analysis of chromosomes 8p and 18q, however, revealed an interesting finding. We did not identify any tumors retaining both 8p and 18q alleles (8pR/18qR, where R = retention), while the other combinations of chromosome 18q and 8p allelic statuses accounted for 20.4% (8pR/18qAI), 35.2% (8pAI/18qR) and 44.4% (8pAI/18qAI) of all tumors (Table 2). Moreover, compared to the 8pR/18qAI group, the 8pAI/18qR group (Odds Ratio = 9, exact p-value = 0.049, 95% CI: 1.17, 69.47) and the 8pAI/18qAI group (Odds Ratio = 6, exact p-value = 0.120, 95% CI: 0.76, 47.22) were more likely to be associated with early distant relapse (Table 2).

## Discussion

As we come to recognize the potential limitations of traditional clinical and pathological predictors for early stage breast cancer recurrence, a greater understanding of genetic and molecular pathogenesis is integral to improving diagnosis, prognosis, and care of breast cancer patients. Studies aimed at identifying predictors for adjuvant therapy would require patient populations that are adjuvant-therapy naïve. Current clinical practice presents a challenge to this, however, because many patients will have received adjuvant therapy before their risk for recurrence can be assessed. In this study, we had an opportunity to study adjuvant therapy naïve patients from Belgium and to examine their tumor characteristics and subsequent disease progression.

Chromosomal instability is the most common alteration seen in breast cancer. Because chromosomes 8p and 18q are frequently deleted in many solid tumors, we thought to evaluate the role of 18q and 8p AI in breast cancer carcinogenesis as these chromosomes could potentially harbor important tumor suppressor genes (TSGs). Although our original samples consisted of both IDC and invasive lobular carcinoma (ILC), we limited our samples to IDC as other studies have observed that IDC and ILC show different profiles in oncogenic amplification as well as in loss of heterozygosity (LOH) [17,18]. For example, a comparative genomic hybridization (CGH) study by Richard *et al*. [19] found that the losses of the 16q and 18q arms were significantly different between IDC and ILC and suggested that different combinations of genetic alterations may reflect differences in evolutionary pathways among distinct morphological subtypes of breast carcinoma.

Nevertheless, there are major obstacles affecting the reliable application of AI analysis to clinical specimens, including normal tissue contamination and various degrees of tissue degradation. Conventional methods for AI analysis, such as microsatellite repeat assay, are not optimally designed for such tissue types, and may introduce various experimental biases. The 'counting alleles' method, on the other hand, is specifically designed for archived, paraffin-embedded samples, which allows researchers to overcome the problems of the currently available techniques and significantly enhances the accuracy of testing for AI in clinical specimens.

Another challenge stems from the characteristics of tumors themselves. The tumor genome is a dynamic, unstable entity. Thus, it is conceivable that among the numerous genetic alterations in tumor cells, only a few events lead to the activation of oncogenes or the inactivation of TSGs, while most are random and non-specific to tumor development. The limitation of AI analysis is that allelic loss is not sensitive enough to differentiate between the true inactivation of TSGs and the random loss of surrogate markers, unless specific locations of TSGs are determined.

Our analysis of 8p and 18q in IDC resulted in a surprising finding that none of the tumor samples retained both 8p and 18q alleles. This is in a striking contrast to studies of colorectal and prostate cancer using the same method [12,13]. In these two studies, approximately 15% of the tumors retained both alleles (Table 3). Thus, our findings lead us to hypothesize that the presence of genetic alterations on 8p or 18q may reflect two alternative mechanisms of generation of IDC.

**Figure 1:**
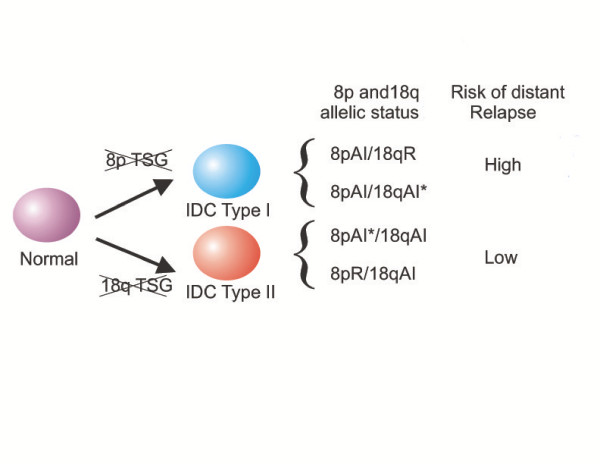
Hypothetical model of tumor subtypes classified by chromosomal 18q and 8p allelic imbalance (AI) status. Inactivation of tumor suppressor genes (TSGs) on either 8p or 18q lead to the formation of two subtypes of invasive ductal carcinoma (IDC) with different risks of distant relapse. Some of the AIs are associated with the inactivation of TSGs, whereas others are non-specific events (denoted by asterisks).

We hypothesized that IDC could be classified into two subtypes based on the critical TSG inactivation on either 18q or 8p (Fig. 1). In addition, we imagined that 18AI/8pAI tumors represent a mixed group of the two subtypes unable to be classified by the theoretical limitation of the AI analysis. In fact, in our analysis, we saw that the 8pAI/18qR group had a higher frequency of distant relapse compared to the 8pR/18qAI group, and the frequency for the 8pAI/18AI group fell between these (Table 2). This phenomenon is unique for IDC because 8pAI/18qAI tumors have the highest risk of recurrence in colorectal cancer in our previous counting allele study [13]. Moreover, the idea of distinct breast carcinogenesis pathways represented by specific allelic losses is also consistent with other CGH and LOH studies, suggesting that there is a preferential combination of genetic alterations. For example, using CGH analysis, Climent *et al*. [20] observed that 8p loss is significantly associated with 8q gain, and 18q loss is significantly associated with 20q gain and 9p loss. The association between 18q loss and 9p loss was also observed by Huiping *et al*. [21]. Therefore, 8p and 18q AI do not share a correlation with the same chromosomal aberrations.

We further thought to address the issue of potential confounders by evaluating the association between the allelic combination and histopathological characteristics. Tumor growth type was the only significantly associated factor in our study population (data not shown). Because the growth type was not significantly associated with distant relapse in our data, nor is there much evidence in the current literature to suggest that it may be a risk factor for distant relapse, only the crude measure of association is reported. In considering potential confounders, we recognized that hormonal status is very important as a prognostic factor of breast cancer. Due to the regional practice standard at the time the samples were collected, the receptor status in many of these patients was not assessed. Nevertheless, we were able to obtain hormonal status information in about 50% of our samples. In our informative samples, there was no association between estrogen receptor status or progesterone receptor status and distant relapse or the allelic status (data not shown).

What are the putative tumor suppressor genes on 8p and 18q whose inactivation can lead to the formation of these two distinct types of IDC? The identification of the TSG on 8p is an active research area in many laboratories, and linkage studies in some breast cancer families that do not have *BRCA1 *and *BRCA2 *mutations suggest that 8p12-22 is a candidate region. Recently, *DBC2 *(deleted in breast cancer 2) was proposed as the candidate tumor suppressor gene on 8p22 because it was homozygously deleted in two breast tumors, and its expression was down-regulated in 50% of sporadic primary breast tumors [22]. More importantly, restoration of its expression in *DBC2*-null breast cancer cell lines led to growth suppression. The biological mechanism of DBC2 function is still under investigation. *MADH4*/*SAMD4*/*DPC4 *is the best candidate for the tumor suppressor gene on 18q because it was homozygously deleted in one out of the eight breast cancer cell lines during the initial screening [23]. Even though no point mutation was identified in *MADH4 *during subsequent screening, immunohistochemistry analysis did indicate that *MADH4 *expression is completely absent in some primary IDCs. Therefore, the genetic/epigenetic inactivation of *MADH4 *in IDC requires careful re-evaluation.

In conclusion, we feel that there may be an interesting association between LOH of 8p and 18q arms in regards to IDC cancer progression. Despite the fact that LOH studies come with both technical and theoretical challenges, we were able to address some of those challenges by using a new quantitative technique to assess a combination of allelic statuses and by limiting our analysis to a more homogenous group of tumors, adjuvant therapy naïve, lymph-node negative IDCs. Because of the relatively small study size, the confidence intervals around our relative odds ratios are quite wide, indicating a substantial level of uncertainty. The interpretation of our findings calls for careful consideration of the study limitations. However, the detected associations are of sufficient magnitude to warrant confirmation in larger studies of the role of 8p and 18q in breast cancer carcinogenesis and progression. If the locations harboring the specific TSGs can be defined, the two subtypes of IDC may be better characterized to have different clinical characteristics and different risks of distant relapse. We thus hope that this unique finding will serve as a hypothesis generating platform for further investigations.

## Conclusion

The present study used a LOH method that was specifically designed for archived specimens, and the initial result suggested the possible presence of two subtypes of early stage IDCs with different potential tumor progression behaviors. Confirmation of this initial finding with a larger data set could provide mechanistic insight into the development of IDCs and may aid the clinical management of patients with IDCs in the future.

## Abbreviations

AI = allelic imbalance; CGH = comparative genomic hybridization; CI = confidence interval; IDC = invasive ductal carcinomas; ILC = invasive lobular carcinoma; LOH = loss of heterozygosity; OR = odds ratio; PCR = polymerase chain reaction; SNP = single-nucleotide polymorphism; TSG = tumor suppressor gene.

## Competing interests

The author(s) declare that they have no competing interests.

## Authors' contributions

AM and TYW contributed equally to this study. AM designed the study and performed the statistical analysis and drafted the manuscript. TYW carried out the 'counting alleles' analysis. CC and LD collected the clinical specimens and patient follow-up information. MG and RHL participated in the design of the study and verified the statistical analysis. DSZ carried out the molecular genetic studies. WZ conceived the study and drafted the manuscript. All authors read and approved the final manuscript.

## Figures and Tables

**Table 1:** Clinical and pathological characteristics in relation to distant relapse

Characteristic	Number of patients	No distant relapse (n)	Distant relapse (n)	p-value
Grade				
1	27	23	4	<0.001
2	20	13	7	
3	18	5	13	
Menopausal status				
Pre	26	15	11	0.46
Post	39	26	13	
Size				
<2 cm	39	29	10	0.02
≥2 cm	26	12	14	
Growth pattern				
Expansive	42	24	18	0.18
Infiltrative	23	17	6	
Necrosis				
No	50	37	13	<0.001
Yes	15	4	11	
Angiogenesis				
Chalkey mean low	35	28	7	0.002
Chalkey mean high	30	13	17	
Fibrotic focus				
No	29	24	5	0.003
Yes	36	17	19	

P-values are based on chi-squared test and Fisher's exact test.

**Table 2:** Allelic status distribution in relation to distant relapse

Allelic type	Number of patients	No distant relapse (n)	Distant relapse (n)	OR	p-value	95% CI
8pAI	47	28	19	Referent	0.18^a^	(0.35, 2.83)
8pR	12	10	2	0.29		
18qAI	40	28	12	Referent	0.19	(0.59, 7.54)
18qR	19	10	9	2.1		
8pR/18qAI	11	10	1	Referent		
8pAI/18qR	19	10	9	9.0	0.049^a^	(1.17, 69.47)
8pAI/18qAI	24	15	9	6.0	0.12^a^	(0.76, 47.22)

^a^P-values based on chi-square test and Fisher's exact test. Approximate 95% confidence intervals (CIs) was calculated for all Odds-ratios. AI, allelic imbalance; R, retention (no allelic imbalance); OR, odds ratio.

**Table 3:** Distribution of 8p/18q allelic imbalance combinations among different cancer types

Allelic type	IDC		CRC^a^		PCA^a^		Exact p-value
	cases	frequency	cases	frequency	cases	frequency	
8pR/18qR	0	0%	27	15%	12	14%	
8pR/18qAI	10	28.5%	25	14%	18	21%	0.03
8pAI/18qR	10	28.5%	35	19%	19	22%	
8pAI/18qAI	15	43%	93	52%	36	42%	

^a^Number of cases and their frequencies for CRC and PCa are based on data from Zhou et al *Lancet *2002, **359:**219–225 and Zhou et al *The Prostate *2004, 61:(1): 81–91. AI, allelic imbalance, R, retention (no allelic imbalance). IDC, Infiltrative ductal carcinoma of breast; CRC, adenocarcinoma of colorectal cancer; PCA, adenocarcinoma of prostate cancer,
